# Six-month longitudinal immune kinetics after mRNA-1273 vaccination: Correlation of peak antibody response with long-term, cross-reactive immunity

**DOI:** 10.3389/fimmu.2022.1035441

**Published:** 2023-01-09

**Authors:** Min Joo Choi, Jung Yeon Heo, Yu Bin Seo, Young Kyung Yoon, Jang Wook Sohn, Ji Yun Noh, Hee Jin Cheong, Woo Joo Kim, Ju-yeon Choi, Hwa Jung Kim, Young Jae Lee, Hye Won Lee, Sung Soon Kim, Byoungguk Kim, Joon Young Song

**Affiliations:** ^1^ Department of Internal Medicine, International St. Mary’s Hospital, Catholic Kwandong University College of Medicine, Incheon, Republic of Korea; ^2^ Department of Infectious Diseases, Ajou University School of Medicine, Suwon, Republic of Korea; ^3^ Division of Infectious Disease, Department of Internal Medicine, Kangnam Sacred Heart Hospital, Hallym University College of Medicine, Seoul, Republic of Korea; ^4^ Department of Internal Medicine, Korea University Anam Hospital, Korea University College of Medicine, Seoul, Republic of Korea; ^5^ Department of Internal Medicine, Korea University Guro Hospital, Korea University College of Medicine, Seoul, Republic of Korea; ^6^ Division of Vaccine Clinical Research, Center for Vaccine Research, National Institute of Infectious Diseases, Cheongju, Republic of Korea; ^7^ Department of Clinical Epidemiology and Biostatistics, ASAN Medical Center, Ulsan University College of Medicine, Seoul, Republic of Korea

**Keywords:** SARS-CoV-2 infection, mRNA-1273 vaccine, COVID-19, cellular immunity, humoral immunity

## Abstract

**Background:**

The emergence of severe acute respiratory syndrome coronavirus 2 (SARS-CoV-2) variants and the persistence of the pandemic, even with mass coronavirus disease 2019 (COVID-19) vaccination, have raised questions about the durability of immunity and extent of cross-reactive immunity after vaccination. This study aimed to characterize the humoral and cellular immune response to the mRNA-1273 vaccine using a prospective longitudinal cohort.

**Methods:**

We recruited 177 young SARS-CoV-2 infection-naive adults. Two doses of mRNA-1273 vaccine were administered at 28-day intervals, and blood samples were collected at five time points: pre-vaccination (T0), 4 weeks after the first (T1) and second dose (T2), and 3 months (T3) and 6 months (T4) after the first dose. Anti-SARS-CoV-2 spike protein (anti-S) IgG antibody, neutralizing antibody, and T-cell immune responses were evaluated.

**Results:**

The two-dose mRNA-1273 vaccination induced robust anti-SARS-CoV-2 antibody responses, which remained higher than the titers at T1 until T4. A higher peak anti-S antibody titer at T2 was associated with better cross-reactive immunity against Delta and Omicron variants and long-lasting (anti-S IgG and neutralizing antibody) humoral immunity up to T4. The overall T-cell immune response was not correlated with peak antibody titers (T-lymphocyte subpopulation analysis was not performed).

**Conclusion:**

This study showed that an early strong antibody response is predictive of longer humoral immunity and better cross-reactive neutralizing immunity against Delta and Omicron variants.

## 1 Introduction

The coronavirus disease 2019 (COVID-19) pandemic continues with more than 600 million confirmed cases and 6.4 million deaths reported worldwide as of August 31, 2022. The administered vaccine dose has reached 12.5 billion as of August 31, 2022 (1), with 67.7% of the world population having received at least one dose of COVID-19 vaccine. In South Korea, 86% of the total population is fully vaccinated with primary series, ranking among the top ten countries worldwide (2). Nevertheless, cases of COVID-19 resurged across countries, particularly in South Korea (2), raising concerns about the longevity of vaccine immunogenicity and immune escape due to the widespread circulation of variants.

The mRNA-1273 vaccine, which displayed a clinical efficacy of 94.5% against confirmed COVID-19 cases in an initial study (3), is the second most commonly used vaccine in South Korea, despite its delayed approval (4, 5). However, there are limited data on cellular immunity and long-term humoral immunity of more than 6 months after mRNA-1273 vaccination (6–11). Booster vaccination (third dose) has been recommended mostly based on the BNT162b2 vaccine data, which showed waning effectiveness and reduced cross-reactive immune responses against severe acute respiratory syndrome coronavirus 2 (SARS-CoV-2) variants (12). However, the mRNA-1273 vaccine demonstrated a stronger immune response in an early study and slower waning of effectiveness compared with that of BNT162b2 (12–14). These results call for long-term follow-up data on immune responses after mRNA-1273 vaccination, particularly in infection-naive individuals.

We previously reported short-term anti-SARS-CoV-2 humoral immunity up to 4 weeks after the second dose of mRNA-1273 vaccination (15). This study aimed to evaluate the longitudinal kinetics of humoral and cellular immunity against SARS-CoV-2 following mRNA-1273 vaccination in young adults. In addition, cross-reactive immunity against variants of concern (VOCs) was also analyzed in a subset of participants.

## 2 Methods

### 2.1 Study design and participants

This multicenter, prospective cohort study was initiated in June 2021, at four university hospitals, near the time that the mRNA-1273 vaccine was approved in South Korea. Healthy adults, aged ≥19-years-old, who were scheduled to receive a two-dose mRNA-1273 vaccination (100 µg/dose) were recruited. Individuals were excluded from the study if they had a prior SARS-CoV-2 infection, autoimmune disease, or immunocompromising conditions. We confirmed that none of the participants were SARS-CoV-2 infected during the study period using nuclear capsid (N) protein antibody testing; the anti-N antibody was measured using the SARS-CoV-2 IgG assay (Abbott Laboratories, Chicago, IL, USA), according to the manufacturer’s protocol.

Demographic information, including age, sex, body mass index (BMI), comorbidities, and history of SARS-CoV-2 infection, was collected from each participant. Blood samples were collected at each scheduled visit as follows: T0 (day of the first dose vaccination), 4 weeks after the first dose (T1), 4 weeks after the second dose (T2), and 3 months (T3) and 6 months (T4) after the first dose. When collecting blood samples at each time point, all participants were checked for SARS-CoV-2 infection since their last visit. In addition, 7 days after each dose of vaccine was administered, the participants were requested to record the presence of solicited adverse events (AEs) through a standardized electronic questionnaire. This study was approved by the ethics committees of the Korea University Guro Hospital (2021GR0099), Ajou University Hospital (AJIRB-BMR-SMP-21-267), Kangnam Sacred Hallym University Hospital (HKS 2021-05-023), and International St. Mary’s Hospital (S21MIME0045). All participants provided written informed consent (Clinical Trial Number - NCT05258708). The trial was conducted under current Good Clinical Practices.

### 2.2 Measurement of immunogenicity

Anti-SARS-CoV-2 spike protein (anti-S) IgG antibodies were assayed using an electrochemiluminescence immunoassay (Elecsys anti-SARS-CoV-2 spike ECLIA, Roche Diagnostics, Pleasanton, CA, USA), according to the manufacturer’s protocol. For the analysis of factors influencing humoral immune responses, a strong antibody response was defined as anti-S IgG antibody titers > 5400 U/mL at the peak period (T2), which is four-fold higher than the IgG titer correlated with a viral neutralization titer ≥160 (16). When we applied the same criteria (>5400 U/mL) in a previous short-term immunogenicity study, only the top third had an anti-S IgG antibody titer > 5400 U/mL (15). The plaque reduction neutralization test (PRNT) was performed using the wild-type (WT) SARS-CoV-2 virus (hCoV/Korea/KCDC03/2020), as described previously (15). The median neutralizing titer (ND_50_) was defined as the concentration of antibodies that reduced the number of viruses by 50%; a threshold ≥ 1:20 was considered positive. The PRNT assay was performed only on samples from the participants at two hospitals. Using age-stratified sampling, we randomly selected a subset of participants. In this subset of participants, we also analyzed cross-neutralizing activity against VOCs, including Delta (B.1.617.2 lineage, hCoV-19/Korea/KDCA229079/2021) and Omicron (lineage B.1.1.529, hCoV-19/Korea/KDCA447321/2021).

In addition, an IFN-γ enzyme-linked immunosorbent spot (ELISpot) assay was performed to quantify SARS-CoV-2-specific cellular immune responses in peripheral blood mononuclear cells (PBMCs) from 45 randomly selected participants at T2 and T3. ELISpot plates (Human IFN-γ ELISpotPRO kit, Mabtech AB, Nacka Strand, Sweden) were blocked with RPMI medium 1640 (Gibco, Grand Island, NY, USA) containing 10% fetal bovine serum (Gibco) and 1% penicillin/streptomycin (Gibco). After washing, the plates were incubated with 2 μg/well of SARS-CoV-2 spike (ID: P0DTC2) peptide pools (GenScript, Piscataway, NJ, USA) and 3 × 10^5^ PBMCs/well. Stimulation with DMSO or PMA/ionomycin was used as negative and positive controls, respectively. The plates were then processed according to the manufacturer’s protocol, and the median spot forming units (SFUs) were counted using the ELISpot reader. The results are presented as SFUs per million input PBMC (SFUs/10^6^ PBMC). The ELISpot assay was also performed only on participants from two hospitals, and some samples were not tested due to the poor PBMC quality.

### 2.3 Statistical analysis

A repeated-measures analysis of variance (ANOVA) was used to evaluate the changes in antibody titers at time points T0–T4 within the group of participants. Log-transformed data were used to calculate the geometric mean titers (GMTs) with 95% confidence intervals (CI). The geometric mean ratio (GMR) was calculated as the mean difference of the measurements on a log scale. The χ^2^ test or Fisher’s exact test was used for categorical variables, whereas the Student’s t-test or one-way ANOVA was used to compare continuous variables, followed by Scheffé’s test for multiple comparisons. Correlations were calculated using Pearson’s correlation coefficient. The results were considered statistically significant at *p*-value < 0.05.

Antibody decay rates and half-life were calculated using an exponential decay model fit to data starting on the study day corresponding to the T2 time point and beyond. Participants who missed even one blood sample collection after the T2 time point were excluded from the decay rate analysis. The exponential model after log_10_ transformation of titers took the following form (17): log_10_ (titer_ij_) = α + β*(study day_ij_) + u_i_ + e_ij_. Where α and β are the intercept and decay rate, respectively; u_i_ is the random intercept for each participant i (considering repeated measures per participant) and e_ij_ is the model error for participant i on study day j. The mixed-effects model was constructed to fit repeated measures with the “proc mixed” procedure. The half-life was computed using the delta method: t_1/2_ = log_10_ (0.5)/β'. Where t_1/2_ is the day on which the titer has decayed to half of its starting value, and β’ is the estimated model decay rate. To compare the half-life between strong and normal responders, a responder variable was included in the model considering repeated measure.

Statistical analyses were performed using SPSS Statistics for Windows, version 24.0 (IBM Corp., Armonk, NY, USA) and SAS for Windows 9.4 software platform (SAS Institute Inc., NC, USA).

## 3 Results

### 3.1 Characteristics of the study population

**Figure 1:**
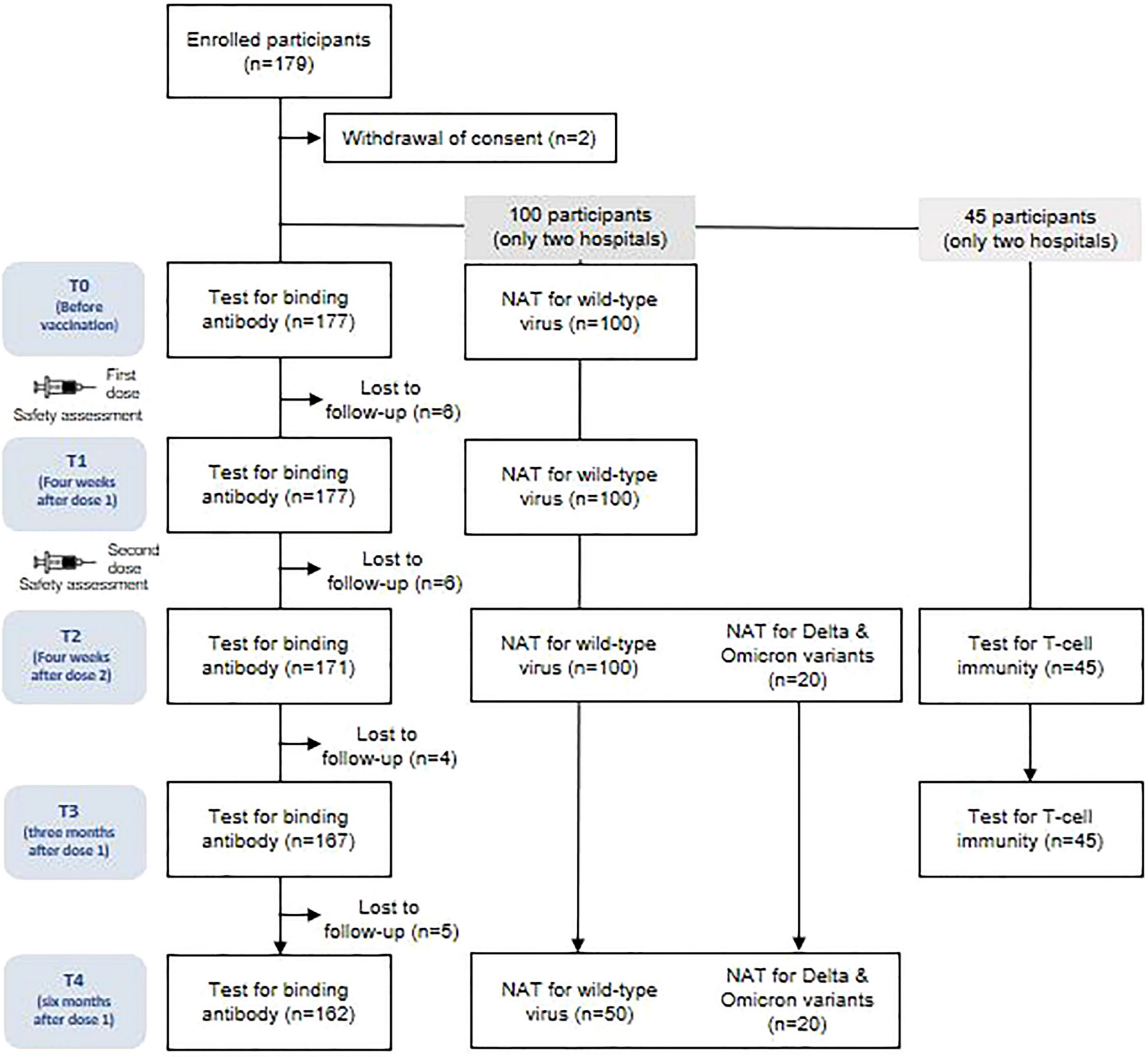
Study diagram. NAT, neutralizing antibody assay.

A total of 177 participants were included in this study. However, owing to missed visits, blood samples were obtained from 162 (91.5%) participants at all time points, 167 (94.4%) at four time points, 171 (96.6%) at three time points, and 177 (100%) at two time points (**Figure 1**). The baseline characteristics of the study participants are presented in **Supplementary Table 1**. The mean age of all participants was 25.4 ± 3.9 (standard deviation, SD) years, with 70% being women. The BMI (mean ± SD) at baseline was 21.6 ± 2.9 kg/m^2^. All of the participants were healthy and had no comorbidities. The interval (mean ± SD) between vaccine doses 1 and 2 was 28.9 ± 2.3 days. The intervals (mean ± SD) from dose 1 to follow-up time points were as follows: 23.7 ± 3.3 days to T1, 56.8 ± 1.8 days to T2, 78.3 ± 4.3 days to T3, and 172.0 ± 9.2 days to T4. The interval (mean ± SD) from dose 2 to T2 was 27.9 ± 3.0 days. None of the participants tested positive for SARS-CoV-2 infection during the study period.

### 3.2 Anti-S IgG and neutralizing antibody responses over time

**Figure 2:**
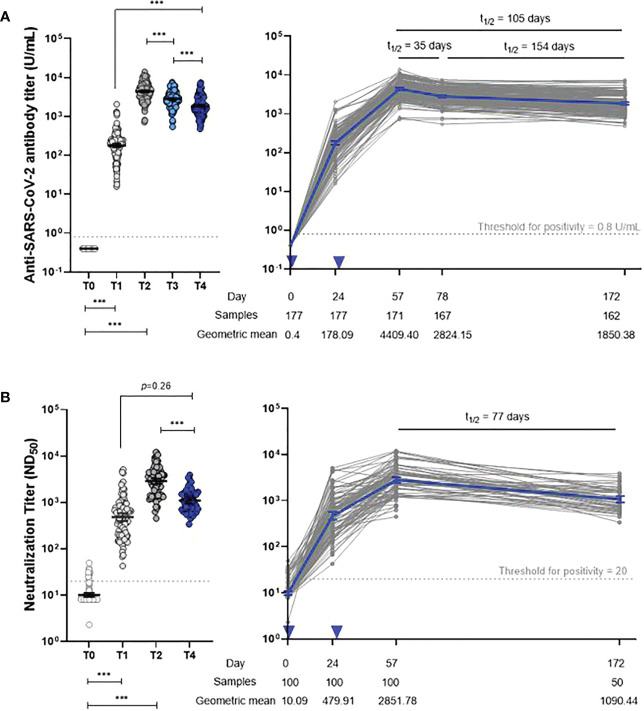
Dynamics of anti-S IgG **(A)** and neutralizing antibody **(B)** responses. Two-tailed p-value resulting from repeated-measures ANOVA with *post-hoc* test (*p < 0.05, **p < 0.01, ***p < 0.001). Error bar depicts mean with 95% confidence interval. Inverted triangle indicates vaccine administration. T0, day of the first dose of vaccine; T1, 4 weeks after the first dose; T2, 4 weeks after the second dose; T3, 3 months after the first dose; T4, 6 months after the first dose.

The anti-S IgG antibody kinetics revealed peak responses at 4 weeks after dose 2 (T2) and progressively decreased over the subsequent 4 months, with a decelerating rate of decline between T2 and T4 (**Figure 2**; **Supplementary Table 2**). However, 6 months after dose 1 (T4), anti-S IgG antibody titers were still significantly higher than those measured 4 weeks after dose 1 (T1). Anti-S IgG antibody titers changed significantly at all study time points (*p* < 0.001). There was a 58% decrease in the average anti-S IgG antibody titer from the peak period (T2) to T4. The half-life of anti-S IgG antibodies was 35 days, 154 days, and 105 days at T2–T3, T3–T4, and T2–T4, respectively.

The neutralizing antibody responses among 100 participants showed kinetics similar to anti-S IgG antibodies; GMTs declined from 2851.8 (2481.9–3276.7) at T2 to 1090.4 (929.5–1279.3) at T4 (**Figure 2**; **Supplementary Table 2**). The neutralizing antibody half-life was 77 days at T2 and beyond.

### 3.3 Cross-reactive neutralizing immunogenicity against variants of concern

**Figure 3:**
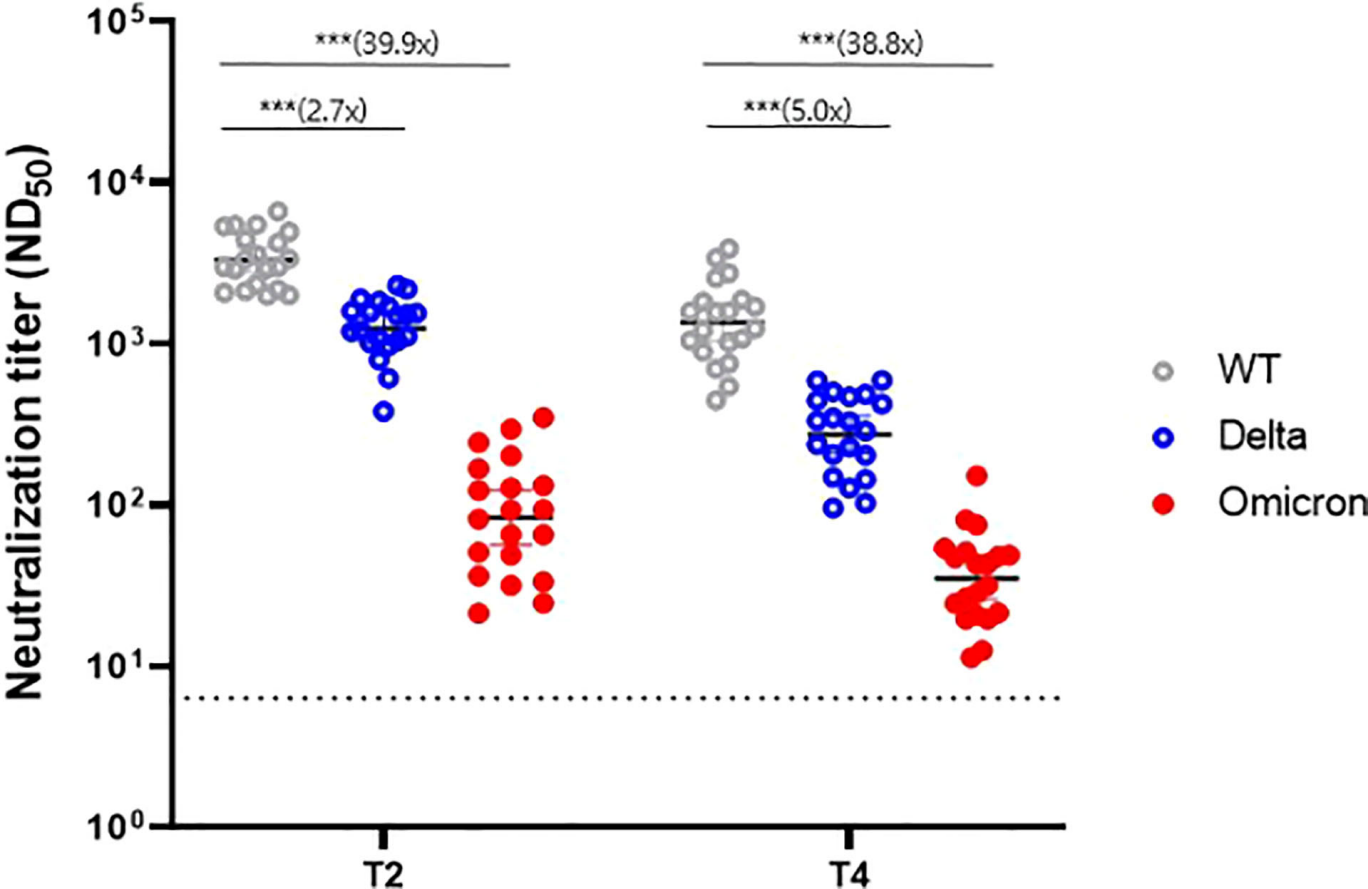
Neutralizing antibody response against variants of concern at two time points (T2 and T4). WT, wild-type. *** denotes *p* < 0.001. T2, 4 weeks after the second dose; T4, 6 months after the first dose.

In a subset of 20 randomly selected participants, PRNT was performed against the WT, Delta variant, and Omicron variant to evaluate cross-reactive immunogenicity. At the peak period (T2) of the humoral immune response, the GMTs were 3324.1 for the WT, 1249.4 for the Delta variant, and 83.4 for the Omicron variant. These values represented a 2.7-fold and 39.9-fold reduction for the Delta and Omicron variants, respectively, compared with the WT (**Figure 3**). At T4, the GMTs were 1353.9 for the WT, 272.1 for the Delta variant, and 34.9 for the Omicron variant, representing a 5.0-fold and 38.8-fold reduction, respectively, compared with the WT. Neutralizing antibodies waned over time; the decline rate was faster in the order of the Delta variant (78%, 70–84%), WT (62%, 52–69%), and Omicron variant (58%, 33–74%) (**Supplementary Figure 1**). Nevertheless, most sera from T4 (6 months after dose 1, average 176 days) still neutralized Delta and Omicron variants in the PRNT (100% and 85%, respectively).

Correlation analysis of neutralization titers between the WT strain and each variant strain revealed a significant correlation for all variants, although the significance disappeared for the Omicron variant at T4 (Pearson’s r = 0.34, *p* = 0.149) (**Supplementary Figure 2**).

### 3.4 SARS-CoV-2-specific T-cell immune response

**Supplementary Figure 3** shows the IFN-γ ELISpot assay results regarding T-cell responses to the SARS-CoV-2 S protein in 45 participants. After the second vaccination dose at T2 and T3, 41 (91%) and 40 (89%) participants showed a positive IFN-γ ELISpot response, with median numbers of SFUs of 66.89 (95% CI, 37.68–118.76) and 24.85 (95% CI, 14.74–41.90), respectively. Unlike the humoral immune response, the temporal trend of individual plots of cellular response was substantially variable, with no significant difference between T2 and T3.

### 3.5 Significance of higher peak anti-S IgG antibody levels on long-term humoral immunity, cross-reactive immunity, and cellular immunity

**Figure 4:**
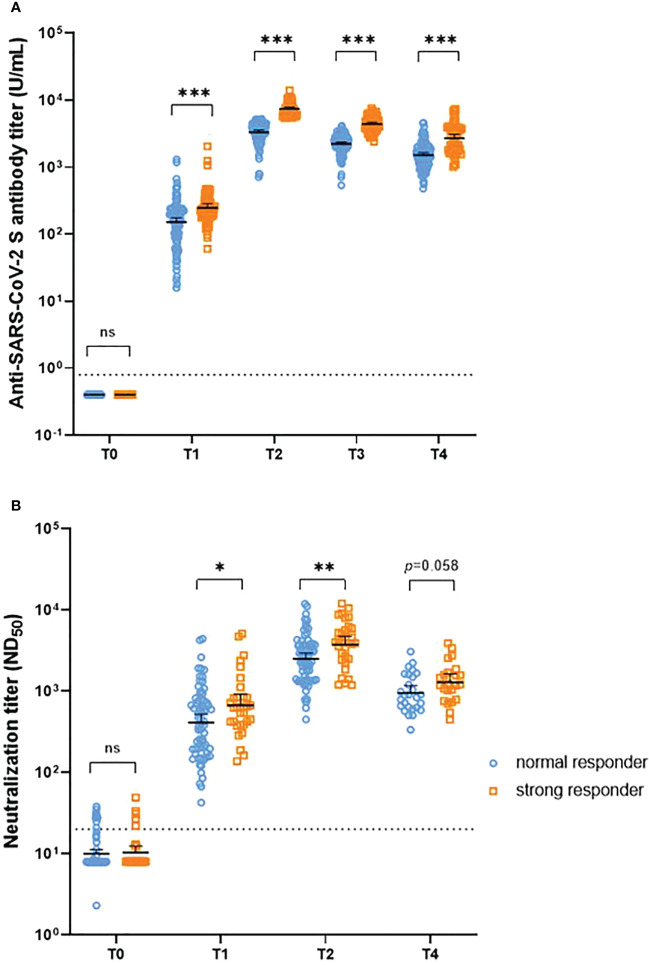
Temporal trend of anti-S IgG antibody titers **(A)** and neutralizing antibody titers **(B)** after vaccination, stratified by the peak anti-S IgG response. NS = *p* > 0.05, *p < 0.05, **p < 0.01, ***p < 0.001. T0, day of the first dose of vaccine; T1, 4 weeks after the first dose; T2, 4 weeks after the second dose; T3, 3 months after the first dose; T4, 6 months after the first dose.

A steeper decline in antibodies was observed in the strong responders (peak anti-S IgG titers >5400 U/mL) than in the normal responders (**Supplementary Table 2**). Between T2 and T4, strong responders showed a 63% and 66% decrease in anti-S IgG and neutralizing antibody titers, respectively, whereas normal responders showed a 54% and 62% decrease, respectively. The half-life of anti-S IgG at T2-T4 was significantly shorter in strong responders compared to normal responders (89 days versus 116 days, p<0.001). In comparison, although statistically insignificant, the half-life of neutralizing antibodies at T2-T4 was longer in strong responders compared to normal responders (85 days versus 70 days, p=0.18). Nevertheless, strong responders still presented higher anti-S IgG antibody titers than normal responders up to 6 months after the first dose (1.78-fold, 1.53–2.08). Regarding the neutralizing antibodies, strong responders showed higher titers than that of normal responders at most time points, although the significance disappeared at T4 (1.35-fold, 0.99–1.85) (**Figure 4**; **Table 1**; and **Supplementary Table 2**). The neutralization activity against Delta or Omicron variants at the peak period was higher in strong responders than in normal responders, but this difference was attenuated at T4. A strong anti-S IgG response was not associated with the magnitude of cellular immunity (**Table 1**).

**Table 1:** Correlation of peak anti-S IgG antibody response with long-term humoral immunity, cross-reactive immunity, and cellular immunity.

	Normal responder	Strong responder	p-value	Ratio of mean titer (95% CI)
Anti-S IgG antibody (T2)	3332.23 (3101.11–3580.57)	7403.23 (6960.30–7874.35)	< 0.001	2.22 (2.02–2.44)
Anti-S IgG antibody (T4)	1521.03 (1394.21–1659.37)	2709.35 (2367.16–3101.01)	< 0.001	1.78 (1.53–2.08)
Neutralizing antibody against WT (T2)	2500.86 (2116.84–2954.54)	3769.64 (2982.86–4763.93)	0.006	1.51 (1.13–2.01)
Neutralizing antibody against WT (T4)	949.18 (767.20–1174.34)	1283.30 (1008.81–1632.47)	0.058	1.35 (0.99–1.85)
Neutralizing antibody against Delta (T2)	826.64 (18.02–37930.79)	1308.09 (1058.90–1615.92)	0.165	1.58 (0.81–3.08)
Neutralizing antibody against Delta (T4)	256.12 (59.76–1097.74)	273.94 (202.12–371.28)	0.728	1.07 (0.68–1.68)
Neutralizing antibody against Omicron (T2)	22.89 (9.17–57.16)	96.30 (66.55–139.35)	0.016	4.21 (1.36–13.04)
Neutralizing antibody against Omicron (T4)	18.17 (0.16–2099.28)	37.50 (27.43–51.27)	0.137	2.06 (0.78–5.48)
Cellular immunity (T2)	58.98 (26.65–117.30)	91.22 (27.910–298.17)	0.803	1.55 (0.43–5.53)
Cellular immunity (T3)	28.38 (14.980–53.76)	17.93 (6.52–49.32)	0.716	0.63 (0.20–2.01)

T0, day of the first dose of vaccine; T1, 4 weeks after the first dose; T2, 4 weeks after the second dose; T3, 3 months after the first dose; T4, 6 months after the first dose.

### 3.6 Correlation between anti-S IgG and neutralizing antibody titers

Notably, a correlation was observed between the anti-S IgG and neutralizing antibody values obtained on a per-sample basis (**Supplementary Figure 4**). However, the variability in anti-S IgG responsiveness was unrelated to the cellular response at each time point (**Supplementary Figure 5**).

## 4 Discussion

In this multicenter prospective study of young SARS-CoV-2 infection-naive adults, mRNA-1273 primary series vaccination induced peak antibody responses approximately 4 weeks after the second dose and declined progressively thereafter. However, even 6 months after the first dose (T4), antibody titers remained higher than those measured 4 weeks after the first dose (T1). Notably, compared with normal responders, strong responders at the peak period (T2) showed rather higher cross-immunity against Delta and Omicron variants at each time point, and sustained higher antibody responses up to 6 months after vaccination. In comparison, the T-cell immune response was not correlated with peak antibody titers. Approximately 90% of the participants showed a positive SARS-CoV-2-specific T-cell immune response after the second dose of vaccine.

Our post-vaccination antibody kinetics were similar to those of SARS-CoV-2 infection with respect to the peak, plateau, and waning patterns: an initial rapid decline followed by a slower decrease (18, 19). The half-lives of anti-S IgG antibodies and neutralizing antibodies calculated in this study are similar to the antibody reduction rate and biphasic decay pattern seen in individuals who recovered from SARS-CoV-2 infection (19–21). The half-life of IgG is approximately 3 weeks; therefore, to maintain detectable circulating antibodies for extended periods, continuous production by plasma cells is needed (22). The biphasic decay curve, also shown in our study, is considered evidence of long-lived plasma cell generation after two-dose vaccination. Long-lived plasma cells have recently been reported in patients who recovered from SARS-CoV-2 infection (23). In an experimental mouse model, mRNA vaccination also induced long-lived plasma and memory B cell responses (24). Clinically, a few studies have reported antibody persistence after mRNA-1273 vaccination (6–10), but only a small number of participants were included (6, 7, 9, 10), and neutralizing antibody assays were not conducted (8, 10). Using a large-scale longitudinal cohort, we added meaningful evidence regarding the durability of antibodies up to 6 months after mRNA-1273 vaccination.

Several SARS-CoV-2 VOCs with multiple spike protein mutations have emerged. This study showed a prominent decline in neutralizing activity against these variants, especially the Omicron variant, which surged in early 2022, and spread worldwide. In agreement with our study, a number of studies have reported a 2- to 4-fold reduction in neutralizing activity against the Delta variant and a 30-fold reduction against the Omicron variant compared with that of the WT SARS-CoV-2 strain at the peak point of antibody response (11, 21, 25). Compared with previous studies (11, 26), mRNA-1273 vaccination in this study demonstrated a less pronounced reduction in cross-reactive neutralization against the variants; most individuals showed detectable neutralizing antibodies against the Delta and Omicron variants within 6 months (100% and 85%, respectively) after vaccination, which could be partly explained by differences in the vaccine composition or formulation and ethnicity (genetic variation) between study populations (11, 26, 27).

Notably, this study showed that a strong anti-S IgG response during the peak period is a predictor of both long-term and cross-reactive immunity against SARS-CoV-2 variants. Strong responders tended to have a faster waning of humoral immunity, as shown in a previous study (28). Nevertheless, strong responders in this study induced higher antibody titers than normal responders, even at 6 months after vaccination. In this study, the half-life of the anti-S IgG antibody titer was shorter in strong responders than in normal responders, but the half-life of the neutralizing antibody titer in strong responders was longer than that in normal responders. In individuals in whom the initial antibody immune response is stronger, neutralizing antibodies are likely to persist for longer, even though the anti-S IgG antibody titer declines rapidly. The higher the peak antibody titers after a two-dose primary series of vaccine, the more likely it is that B cells differentiate into high-quality long-lived plasma cells, which would contribute to maintaining the circulating antibody for a longer time.

Vaccine effectiveness against SARS-CoV-2 infection seemed to decrease more slowly than expected through humoral immunogenicity studies, showing a reduction of less than 30% of the peak level in the first 6 months (29, 30), whereas protection against hospitalization and death appeared to be much more robust, with no evidence of waning for several months after the second dose (29). T-cell immunity may contribute to prolonged effectiveness in preventing severe diseases. Regarding T-cell immunity, compared with the rapid decline in specific IgG titers, long-lasting memory T-cells were detected up to 17 years after SARS-CoV infection (31). A previous study on cellular immunity after mRNA-1273 vaccination showed a similar cellular immune response (86% positivity at 3 months after the second dose) with our results; however, they presented a close relationship between humoral and cellular immune responses, contrary to our study (28). Another study revealed that T-cell responses after the first dose, which was not measured in our study, correlated with antibodies at 6 months, highlighting a key role for early CD4 T-cell responses (9). In this study, cellular immunity was not correlated with humoral immunity at any time point, which might be related to the differences in the study population with respect to age and pre-existing cross-reactive T-cell immunity from seasonal coronavirus infections (32). Alternatively, it might be because a T-lymphocyte subpopulation analysis was not conducted, and T-cell immunity was not measured at an early time point.

This study has several limitations. First, it was conducted on healthy young adults, excluding older adults and chronically ill patients. Second, neutralization titers and cellular immunity were measured only in a subset of participants; the small sample size might cause a statistically insignificant results in our analyses. Third, T-cell immunity at the beginning of vaccination might play an important role in the subsequent immune response after vaccination, but the immune response was not measured during the early stage (within 7 days after vaccination). Finally, due to the wide intervals between blood sampling points, the formula for estimating half-life might be somewhat imprecise, so caution is needed in interpretation.

In conclusion, this study showed that an early strong antibody response is predictive of longer humoral immunity and better cross-reactive neutralizing immunity against Delta and Omicron variants. A positive SARS-CoV-2 specific T-cell immune response was induced in most mRNA-1273 vaccine recipients.

## Data availability statement

The original contributions presented in the study are included in the article/**Supplementary Material**. Further inquiries can be directed to the corresponding authors.

## Ethics statement

The studies involving human participants were reviewed and approved by Korea University Guro Hospital (2021GR0099), Ajou University Hospital (AJIRB-BMR-SMP-21-267), Kangnam Sacred Hallym University Hospital (HKS 2021-05-023), and International St. Mary’s Hospital (S21MIME0045). The patients/participants provided their written informed consent to participate in this study.

## Author contributions

MJC, BK, and JYS conceived and designed the study. MJC, JYH, YKY, JWS, YBS, JYN, HJC, WJK, JYC, YJL, HWL, SSK, BK, and JYS contributed to the acquisition of the clinical and laboratory data. All authors contributed to data interpretation. MJC, HJK, JYH, YBS, and JYS contributed to the statistical analysis. MJC and JYS analyzed the data, took responsibility for its integrity, and prepared the manuscript. All the authors reviewed the manuscript for intellectual content and approved the final version for submission.
