# Supplementary material for: Six-month longitudinal immune kinetics after mRNA-1273 vaccination: Correlation of peak antibody response with long-term, cross-reactive immunity
Source: Front Immunol. 2023 Jan 9;13:1035441. doi: 10.3389/fimmu.2022.1035441 (PMC9868900; doi:10.3389/fimmu.2022.1035441)
Supplement: Supplementary file 1 [file DataSheet_1.docx]

**Supplementary Table 1. Baseline characteristics and solicited adverse events after each vaccine dose**

| **Baseline characteristics of all participants** | **T0**  **(n=177)** | **T1**  **(n=177)** | **T2**  **(n=171)** | **T3**  **(n=167)** | **T4**  **(n=162)** |
| --- | --- | --- | --- | --- | --- |
| Age, mean ± SD (range) | 25.4 ± 3.88 (21-55) | 25.4 ± 3.88 (21-55) | 25.5 ± 3.86 (21-55) | 25.6 ± 3.90 (21-55) | 25.5 ± 3.94 (21-55) |
| Male, number (%) | 53 (31.0%) | 53 (31.0%) | 53 (31.0%) | 53 (31.7%) | 51 (31.5%) |
| BMI, mean ± SD | 21.58 ± 2.88 | 21.58 ± 2.88 | 21.6 ± 2.88 | 21.6 ± 2.90 | 21.6 ± 2.87 |
| **AE experienced after each dose** | Dose 1  (n=177) | Dose 2  (n=177) |  |  |  |
| Any AE, number (%) | 176 (99.4%) | 168 (98.2%) |  |  |  |
| Any systemic AE, number (%) | 155 (87.6%) | 163 (95.3%) |  |  |  |
| Fever | 38 (21.5%) | 125 (73.1%) |  |  |  |
| Any local AE, number (%) | 174 (98.3%) | 164 (95.9%) |  |  |  |
| Antipyretic use, number (%) | 107 (60.5%) | 155 (90.6%) |  |  |  |
| **Baseline characteristics of participants who measured neutralization activity** | **T0-T2**  **(n=100)** | | |  | **T4**  **(n=50)** |
| Age, mean ± SD (range) | 26.2 ± 4.43 (22-55) | | |  | 25.8 ± 4.07 (22-49) |
| Male, number (%) | 23 (23.0%) | | |  | 3 (6.0%) |
| BMI, mean ± SD | 21.13 ± 2.78 | | |  | 20.65 ± 2.42 |
| **Baseline characteristics of participants who measured neutralization against VOCs (n=20) at T2 and T4** | | | | | |
| Age, mean ± SD (range) | 27.2 ± 5.57 (23-49) | | | | |
| Male, number (%) | 1 (5.0%) | | | | |
| BMI, mean ± SD | 19.78 ± 2.08 | | | | |
| **Baseline characteristics of participants who measured cellular immunity (n=45) at T2 and T3** | | | | | |
| Age, mean ± SD (range) | 25.6 ± 2.73 (22-32) | | | | |
| Male, number (%) | 14 (31.1%) | | | | |
| BMI, mean ± SD | 21.92 ± 2.90 | | | | |

T0, day of first-dose vaccination; T1, 4 weeks after the first dose; T2, 4 weeks after the second dose; T3, 3 months after the first dose; T4, 6 months after the first dose.

**Supplementary Table 2. Anti-S IgG and neutralizing antibody titers of participants following vaccination**

| Characteristics | Anti-S IgG antibody, U/mL, mean (95% CI) | | | | | | Neutralizing antibody, mean (95% CI) | | | | |
| --- | --- | --- | --- | --- | --- | --- | --- | --- | --- | --- | --- |
|  | T0 | T1 | T2 (peak) | T3 | T4 | GMR (T4/T2) | T0 | T1 | T2 (peak) | T4 | GMR (T4/T2) |
| All | N=177 | N=177 | N=171 | N=167 | N=162 | 0.42 (0.37- 0.47) | N=100 | N=100 | N=100 | N=50 | 0.38 (0.31–0.48) |
|  | 0.4 (0.4–0.4) | 178.09 (159.0–199.46) | 4409.40 (4082.56– 4762.40) | 2824.15 (2633.06– 3029.11) | 1850.38 (1700.91– 2012.98) |  | 10.09 (9.13–11.16) | 479.91 (394.37– 584.01) | 2851.78 (2481.94– 3276.73) | 1090.44 (929.49– 1279.25) |  |
| Responder | | | | | | | | | | | |
| Normal | N=111 | N=111 | N=111 | N=109 | N=107 | 0.46 (0.41- 0.51) | N=68 | N=68 | N=68 | N=27 | 0.38 (0.28– 0.51) |
|  | 0.4 (0.4–0.4) | 152.04 (130.80– 176.72) | 3332.23 (3101.11– 3580.57) | 2230.32 (2087.17– 2383.30) | 1521.03 (1394.21– 1659.37) |  | 9.98 (8.83– 11.28) | 410.13 (321.10– 523.84) | 2500.86 (2116.84– 2954.54) | 949.18 (767.20– 1174.34) |  |
| Strong | N=60 | N=60 | N=60 | N=58 | N=55 | 0.37 (0.32- 0.42) | N=32 | N=32 | N=32 | N=23 | 0.34 (0.24–0.48) |
|  | 0.4 (0.4–0.4) | 247.04 (212.70– 286.92) | 7403.23 (6960.30– 7874.35) | 4401.05 (4096.76– 4727.94) | 2709.35 (2367.16– 3101.01) |  | 10.34 (8.59– 12.45) | 670.15 (490.67– 915.30) | 3769.64 (2982.86– 4763.93) | 1283.30 (1008.81– 1632.47) |  |
| P-value | 1.000 | <0.001 | <0.001 | <0.001 | <0.001 |  | 0.746 | 0.020 | 0.006 | 0.058 |  |

GMR, geometric mean ratio. T0, day of first-dose vaccination; T1, 4 weeks after the first dose; T2, 4 weeks after the second dose; T3, 3 months after the first dose; T4, 6 months after the first dose.

**Supplementary Figure 1.** **Spaghetti plot of temporal change of neutralizing activity against (A) delta and (B) omicron variants.** T2, 4 weeks after the second dose; T4, 6 months after the first dose.


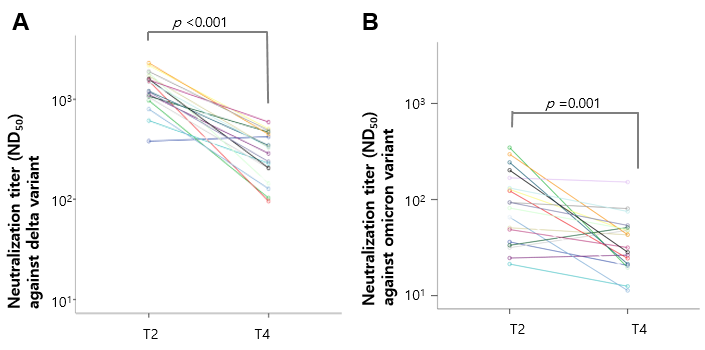


**Supplementary Figure 2. Correlation analysis between neutralization titers of wild type versus delta or omicron at T2 (A) and T4 (B).** T2, 4 weeks after the second dose; T4, 6 months after the first dose.


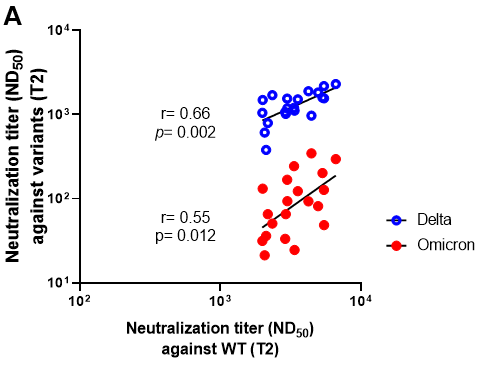

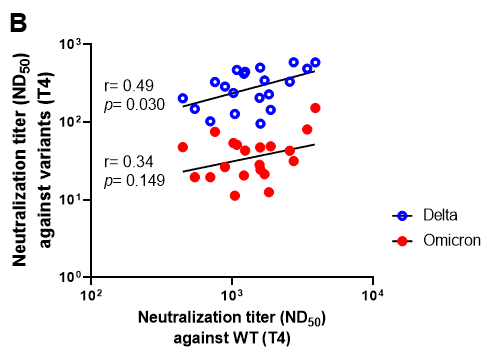


**Supplementary Figure 3.** IFN-γ ELISpot assay results at two time-points (T2 and T3). T2, 4 weeks after the second dose; T3, 3 months after the first dose.


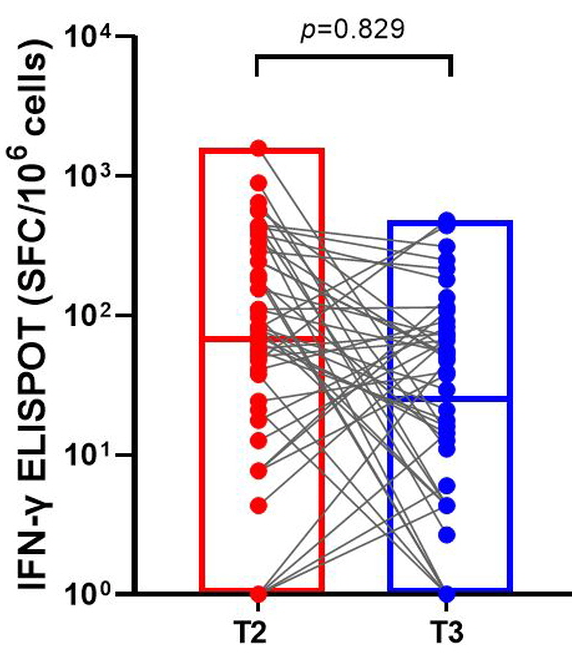


**Supplementary Figure 4. Correlation analysis between anti-S IgG antibody and neutralizing antibody titers.** T1, 4 weeks after the first dose; T2, 4 weeks after the second dose; T4, 6 months after the first dose.

**Supplementary Figure 5. Correlation analysis between anti-S IgG antibody titers and IFN-γ ELISpot assay results.** T2, 4 weeks after the second dose; T3, 3 months after the first dose
